# Loss of Function of the Cik1/Kar3 Motor Complex Results in Chromosomes with Syntelic Attachment That Are Sensed by the Tension Checkpoint

**DOI:** 10.1371/journal.pgen.1002492

**Published:** 2012-02-02

**Authors:** Fengzhi Jin, Hong Liu, Ping Li, Hong-Guo Yu, Yanchang Wang

**Affiliations:** 1 Department of Biomedical Sciences, College of Medicine, Florida State University, Tallahassee, Florida, United States of America; 2 Department of Biological Science, Florida State University, Tallahassee, Florida, United States of America; The University of North Carolina at Chapel Hill, United States of America

## Abstract

The attachment of sister kinetochores by microtubules emanating from opposite spindle poles establishes chromosome bipolar attachment, which generates tension on chromosomes and is essential for sister-chromatid segregation. Syntelic attachment occurs when both sister kinetochores are attached by microtubules from the same spindle pole and this attachment is unable to generate tension on chromosomes, but a reliable method to induce syntelic attachments is not available in budding yeast. The spindle checkpoint can sense the lack of tension on chromosomes as well as detached kinetochores to prevent anaphase onset. In budding yeast *Saccharomyces cerevisiae*, tension checkpoint proteins Aurora/Ipl1 kinase and centromere-localized Sgo1 are required to sense the absence of tension but are dispensable for the checkpoint response to detached kinetochores. We have found that the loss of function of a motor protein complex Cik1/Kar3 in budding yeast leads to syntelic attachments. Inactivation of either the spindle or tension checkpoint enables premature anaphase entry in cells with dysfunctional Cik1/Kar3, resulting in co-segregation of sister chromatids. Moreover, the abolished Kar3-kinetochore interaction in *cik1* mutants suggests that the Cik1/Kar3 complex mediates chromosome movement along microtubules, which could facilitate bipolar attachment. Therefore, we can induce syntelic attachments in budding yeast by inactivating the Cik1/Kar3 complex, and this approach will be very useful to study the checkpoint response to syntelic attachments.

## Author Summary

Chromosome bipolar attachment occurs when sister chromatids are attached by microtubules emanating from opposite spindle poles and is essential for faithful sister-chromatid segregation. Chromosomes are under tension once bipolar attachment is established. The absence of tension is sensed by the tension checkpoint that prevents chromosome segregation. The attachment of sister chromatids by microtubules from the same spindle pole generates syntelic attachment, which fails to generate tension on chromosomes. However, a reliable method to induce syntelic attachment is not available. Our findings indicate that the inactivation of the motor complex, Cik1/Kar3, results in chromosomes with syntelic attachment in budding yeast. In the absence of the tension checkpoint, yeast cells with dysfunctional Cik1/Kar3 enter anaphase, resulting in co-segregation of sister chromatids. Therefore, with this method we can experimentally induce syntelic attachment in yeast and investigate how cells respond to this incorrect attachment.

## Introduction

One of the most important events during the cell cycle is chromosome segregation and errors in this process will lead to chromosome missegregation. To separate sister chromatids into daughter cells, sister kinetochores must be attached to the microtubules emanating from opposite spindle poles in order to establish bipolar attachment. Even though this process is highly regulated, incorrect attachment takes place occasionally. Syntelic attachment occurs when both sister kinetochores are connected to microtubules from the same spindle pole. In monotelic attachment, only one of the sister kinetochores connects to the microtubules from a spindle pole [1]. It is also possible for both sister kinetochores to be detached. These incorrect attachments have to be corrected before anaphase entry, or chromosome missegregation will occur.

The kinetochore is a multi-protein complex that connects chromosomes to microtubules. More than 60 kinetochore proteins have been identified in budding yeast. The CBF3 (centromere binding factor) complex associates directly with centromeric DNA, while the DASH/Dam1 complex residues at the kinetochore-microtubule interface. As a ten-protein complex including Dam1 and Ask1, the DASH can form a ring structure around a single microtubule and mediate the kinetochore-microtubule interaction [2], [3], [4], [5]. Ndc80 (Ndc80, Nuf2, Spc24, Spc25), COMA (Ctf19-Okp1-Mcm21-Ame1), and MIND (Mtw1p including Nnf1-Nsl1-Dsn1) complexes bridge the gap between centromere-bound CBF3 and microtubule-associated DASH [6], [7].

Chromosome attachment is monitored by the spindle checkpoint which includes Bub1, Bub3, Mad1, Mad2, Mad3, and Mps1 [8], [9], [10], [11]. Detached kinetochores activate the checkpoint by allowing the formation of a Mad2-Mad3/BubR1-Bub3-Cdc20 complex. Because Cdc20 is an essential activator of the anaphase-promoting complex (APC), the binding of Cdc20 by the spindle checkpoint components blocks APC^Cdc20^ activity [12], [13]. APC^Cdc20^ mediates the ubiquitination and the subsequent degradation of the anaphase inhibitor securin, known as Pds1 in budding yeast [14]. Pds1 protein inhibits anaphase by binding to separase Esp1 and preventing Esp1-dependent cleavage of cohesin, a protein complex that holds sister chromatids together [15], [16]. Therefore, the activation of the spindle checkpoint prevents anaphase entry by blocking Pds1 degradation, and stabilized Pds1 protein indicates the activation of the spindle checkpoint.

Chromosome bipolar attachment generates tension on sister kinetochores. The observation that the application of tension on an improperly attached chromosome in grasshopper cells abolishes the anaphase entry delay directly demonstrates the role of tension in cell cycle regulation [17]. To analyze the response to the absence of tension in yeast cells, tension defects can be induced by the block of DNA synthesis or by the abrogation of sister chromatid cohesion [18], [19]. In both situations, the lack of tension prevents anaphase entry as indicated by the stabilized Pds1 protein levels. Ipl1 and Sgo1 were found to be required to sense tension defects and prevent anaphase entry, but they are dispensable for cell cycle arrest induced by the disruption of the spindle structure [19], [20]. In addition to its checkpoint function, Ipl1 kinase also promotes the turnover of kinetochore-microtubule interaction when tension is absent [21], [22]. Therefore, it is speculated that Ipl1 may activate the checkpoint by generating detached chromosomes when tension is absent. In contrast, Sgo1 does not play a role in destabilizing kinetochore attachment and its checkpoint function remains unclear at the molecular level [22].

As one of the six kinesin-related proteins in budding yeast, Kar3 was identified as being essential for yeast nuclear fusion during mating [23]. Unlike other kinesins, Kar3 protein contains a motor domain at its carboxy terminus that possesses minus-end-directed motility [24]. Recent evidence indicates that Kar3 localizes at the spindle midzone and may also function as an interpolar-microtubule cross-linker to prevent spindle collapse [25]. Moreover, Kar3 protein promotes the poleward transport of chromosomes along astral microtubules [26], [27]. Two proteins, Cik1 and Vik1, associate with Kar3 through coiled-coil domains to form Cik1/Kar3 or Vik1/Kar3 heterodimers. Both *kar3Δ* and *cik1Δ* mutants show defects in mating, spindle morphogenesis, and chromosome segregation [28], but their direct role in mitosis remains unclear.

We previously showed that *cik1Δ* and *kar3Δ* mutants are sensitive to hydroxyurea (HU), a DNA synthesis inhibitor, and these mutants exhibit chromosome bipolar attachment defects after HU treatment [29]. We recently found that *cik1Δ* and *kar3Δ* mutants are synthetically lethal with tension checkpoint mutants *ipl1-321* and *sgo1Δ*, indicating a role for Cik1/Kar3 in chromosome segregation. To further study the function of Cik1/Kar3, we constructed a plasmid *P_GAL_CIK1-CC* that contains the coiled-coil domain of Cik1. Our results indicate that overexpression of *CIK1-CC* can competitively disrupt the Cik1-Kar3 interaction, which allows us to conditionally abolish Cik1/Kar3 function. With this method, we show that dysfunctional Cik1/Kar3 results in significant co-segregation of sister chromatids in the absence of the spindle checkpoint. Strikingly, dysfunctional Cik1/Kar3 also causes co-segregation of sister chromatids in *ipl1-321* and *sgo1Δ* cells. Given the role of Ipl1 and Sgo1 in sensing chromosomes that lack tension, these data suggest that the loss of function of Cik1/Kar3 results in an increased frequency of syntelic attachment. Results with live-cell imaging and cohesin mutants further support this conclusion. Therefore, syntelic attachments can be induced in budding yeast by inactivating Cik1/Kar3 complex and this method will be a very useful tool for studying the response to tension defects.

## Results

### Overexpression of *CIK1-CC* mimics the phenotype of *cik1Δ* and *kar3Δ*

Our previous study indicates that the Cik1/Kar3 complex facilitates chromosome bipolar attachment after treatment with HU, an inhibitor of DNA synthesis [29]. We also noticed that *cik1Δ* and *kar3Δ* mutants exhibited an anaphase entry delay in the absence of HU, suggesting the presence of improper chromosome attachments. Previous work shows that both *cik1* and *kar3* mutants are synthetically lethal with spindle checkpoint mutants, *bub1*, *mad1*, *mad2*, and *mad3* [30], [31]. Interestingly, we found that *cik1Δ* and *kar3Δ* are also synthetically lethal with tension checkpoint mutants *sgo1Δ* and *ipl1-321*. This genetic interaction with tension checkpoint mutants suggests that the Cik1/Kar3 complex may facilitate the establishment of chromosome bipolar attachment that generates tension on chromosomes.

To further study the role of Cik1/Kar3 in chromosome bipolar attachment, we need to examine chromosome segregation in *cik1Δ* and *kar3Δ* mutants in the absence of the spindle checkpoint, which allows anaphase entry in spite of incorrect chromosome attachments. Because of the synthetic lethality, we have to develop a way to conditionally inactivate the Cik1/Kar3 complex. Kar3 and Cik1 associate with each other through their respective coiled-coil domains [32], thus overexpression of this domain may competitively disrupt the Cik1-Kar3 interaction. We constructed a plasmid *P_GAL_CIK1-CC* that contains the coiled-coil domain of *CIK1* under control of a galactose inducible promoter and the Cik1-Kar3 interaction in cells overexpressing *CIK1-CC* was examined.

**Figure 1:**
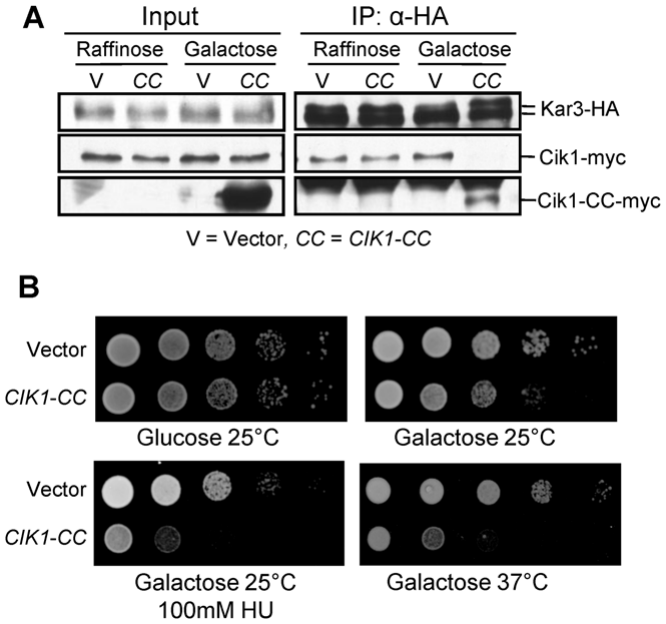
Overexpression of *CIK1-CC* disrupts the Cik1–Kar3 interaction and phenocopies the growth defects of *cik1* and *kar3* mutants. A. Overexpression of *CIK1-CC* abolishes Cik1-Kar3 interaction. *CIK1-13myc KAR3-3HA* cells with a vector (V) or a *P_GAL_CIK1-CC* (*CC*) plasmid were grown to mid-log phase in raffinose medium at 30°C. After galactose was added to the cell cultures for 3 hrs, the cells were collected for immunoprecipitation (IP) with anti-HA antibody and the precipitates were subjected to Western blotting with anti-HA and anti-myc antibodies. B. Overexpression of *CIK1-CC* phenocopies *cik1Δ* and *kar3Δ* mutants. Cell cultures in stationary phase with indicated genotypes were 10-fold diluted and spotted onto glucose or galactose plates with or without 100 mM HU. The plates were scanned after incubation at 25°C or 37°C for 3–4 days.

Consistent with a previous report [28], we detected the interaction between Cik1 and Kar3 in control cells by co-immunoprecipitation. However, the Cik1-Kar3 interaction was completely abolished after *CIK1-CC* overexpression. Instead, the association of Kar3 with the coiled-coil domain of Cik1 (Cik1-CC) was detected, suggesting that the disruption of Cik1-Kar3 interaction by Cik1-CC is in a competitive manner (Figure 1A). Kar3 is able to form a heterodimer with either Cik1 or Vik1 [33], [34]. We also noticed that Vik1-Kar3 interaction was decreased in cells overexpressing *CIK1-CC* (Figure S1). Next we examined the phenotypes of cells overexpressing *CIK1-CC* and found that these cells grew slowly and were sensitive to HU. In addition, cells overexpressing *CIK1-CC* failed to grow at 37°C (Figure 1B), which is reminiscent of *cik1Δ* and *kar3Δ* mutants [29]. Therefore, we conclude that overexpression of *CIK1-CC* disrupts Cik1-Kar3 interaction and cells overexpressing *CIK1-CC* mimic the phenotypes of *cik1Δ* and *kar3Δ* mutants.

### Tension checkpoint mutants abolish the anaphase entry delay induced by *CIK1-CC* overexpression

**Figure 2:**
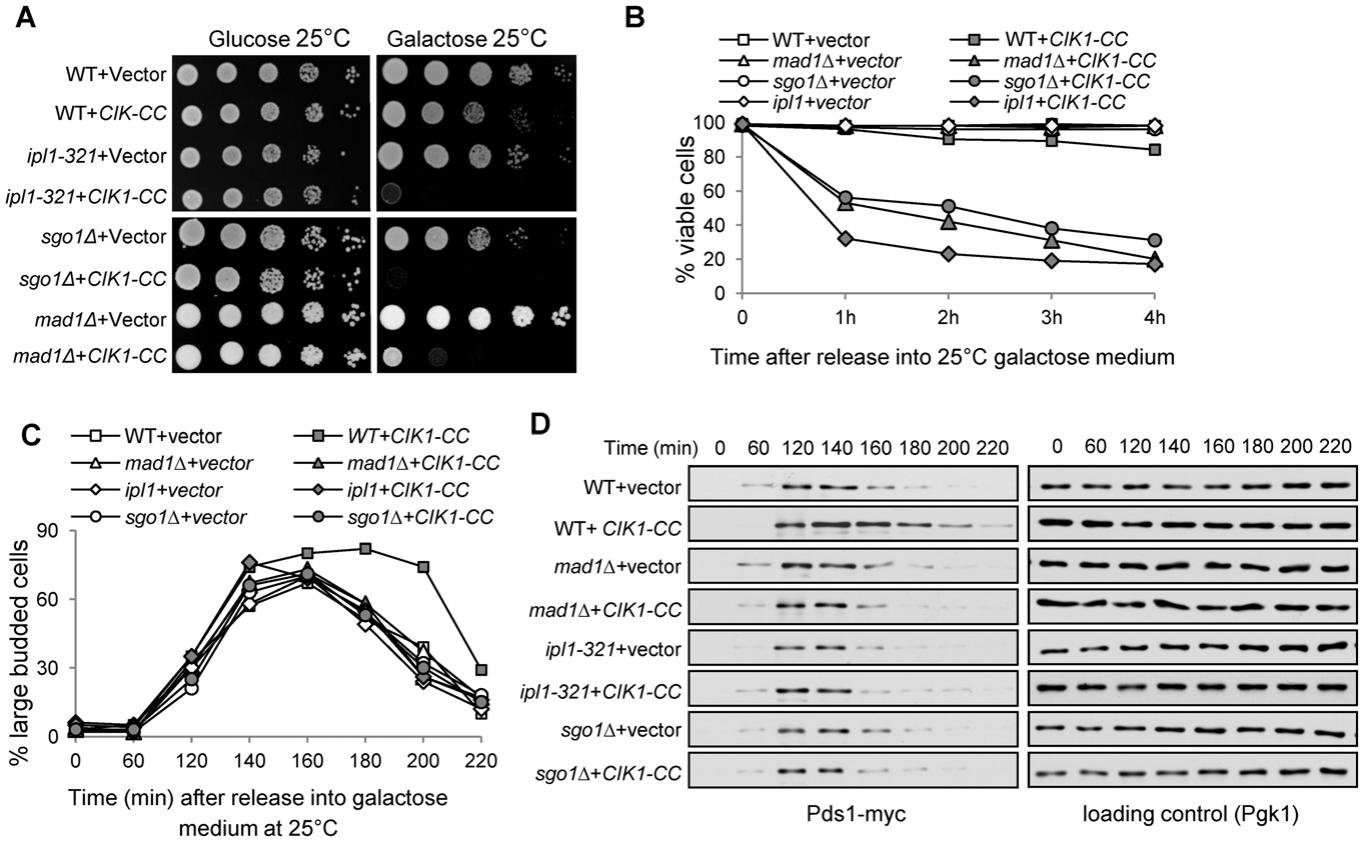
Overexpression of *CIK1-CC* results in checkpoint-dependent anaphase entry delay. A. Overexpression of *CIK1-CC* is lethal to *mad1Δ*, *ipl1-321*, and *sgo1Δ* mutants. Serial 10-fold dilutions of WT and mutant cells with a vector or a *P_GAL_CIK1-CC* plasmid were spotted onto glucose and galactose plates and incubated for 3 days at 25°C. B. *mad1Δ*, *ipl1-321*, and *sgo1Δ* mutant cells lose viability after *CIK1-CC* overexpression. G_1_-arrested cells with the indicated genotypes were released into galactose medium and incubated at 25°C. Cells were collected at the indicated time points and spread onto YPD plates. After overnight growth, the plating efficiency was determined and the percentage of viable cells is shown. C and D. Checkpoint mutants alleviate the Pds1 degradation delay induced by *CIK1-CC* overexpression. G_1_-arrested WT, *mad1Δ*, *ipl1-321*, and *sgo1Δ* cells containing Pds1-18myc as well as a vector or a *P_GAL_CIK1-CC* plasmid were released into galactose medium and incubated at 25°C. α-factor was restored after budding to block the second round of cell cycle. Cells were collected at the indicated time points and protein samples were prepared for Western blotting. The budding index is shown in C and Pds1 levels are shown in D. Pgk1 protein levels were used as a loading control.

Next we examined the growth of the spindle checkpoint mutant *mad1Δ* and tension checkpoint mutants *ipl1-321* and *sgo1Δ* after *CIK1-CC* overexpression. These mutant cells harboring a vector grew well on galactose plates, but the mutant cells with *P_GAL_CIK1-CC* plasmids failed to grow on galactose plates (Figure 2A). Moreover, the checkpoint mutant cells lost viability shortly after the induction of *CIK1-CC* (Figure 2B). Surprisingly, *ipl1-321* mutants with *P_GAL_CIK1-CC* plasmids did not grow on galactose plates and lost viability after incubation in galactose medium at the permissive temperature 25°C (Figure 2A and 2B), which may be due to the fact that mutated Ipl1-321 protein shows significantly impaired kinase activity even at 25°C [35], [36].

We speculate that the slow growth phenotype in cells overexpressing *CIK1-CC* is attributed to the incorrect chromosome attachments that delay anaphase entry by stabilizing Pds1. Moreover, this delay likely depends on the spindle checkpoint. To test this idea, we compared the cell cycle progression in wild-type (WT), *mad1Δ*, *sgo1Δ*, and *ipl1-321* cells after *CIK1-CC* overexpression. After G_1_ release into 25°C galactose medium for 200 min, 39% of WT cells with a control vector were large budded, while the percentage of large budded cells increased to 74% in those expressing *CIK1-CC* (Figure 2C). Consistent with the cell cycle delay, *CIK1-CC* overexpression also caused Pds1 protein stabilization. Strikingly, the cell cycle delay and Pds1 stabilization were eliminated not only in the spindle checkpoint mutant *mad1Δ*, but also in tension checkpoint mutants *sgo1Δ* and *ipl1-321*, which only detect lack of tension (Figure 2D). This result suggests that tension defects but not detached chromosomes activate the checkpoint in cells lacking Cik1/Kar3.

### Cells overexpressing *CIK1-CC* exhibit a chromosome bipolar attachment defect

**Figure 3:**
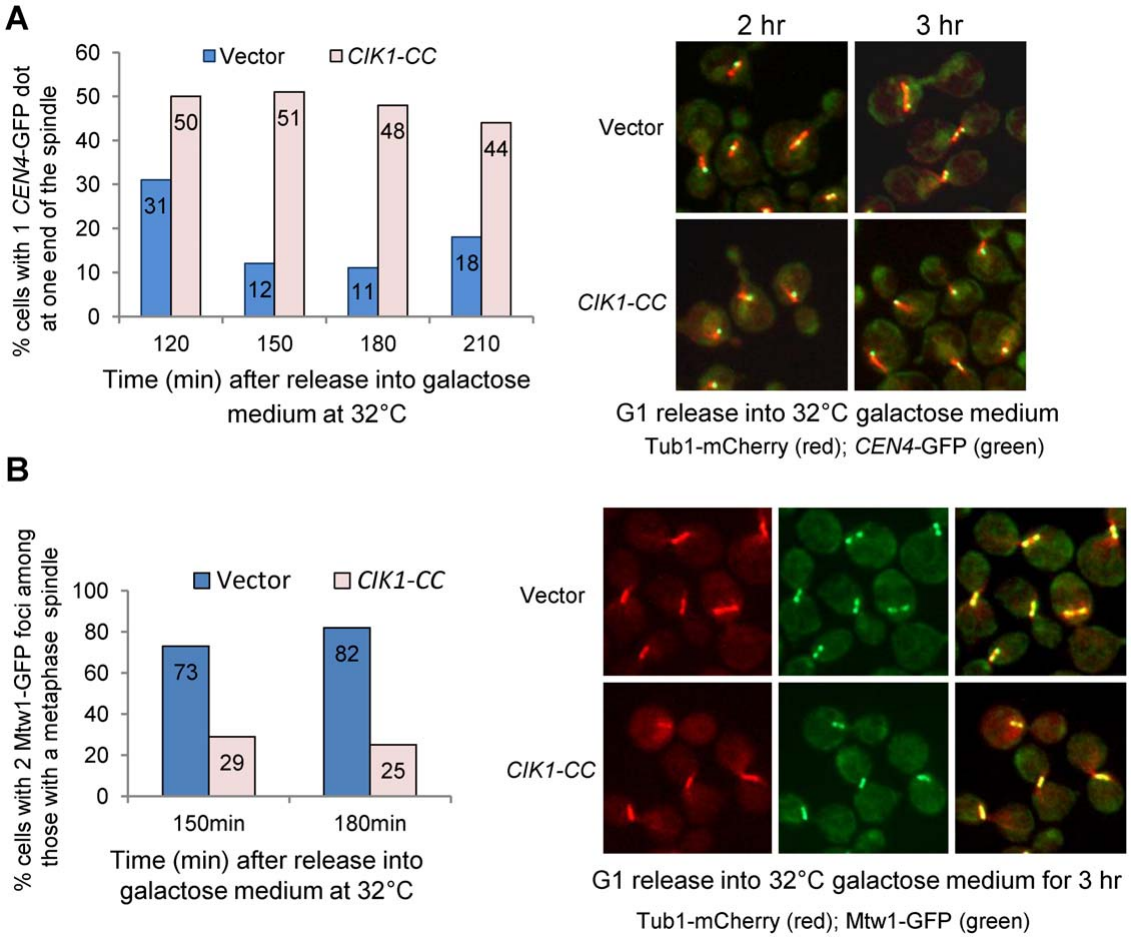
Overexpression of *CIK1-CC* leads to defects in chromosome bipolar attachment. A. G_1_-arrested *cdc13-1 CEN4-GFP TUB1-mCherry* cells with a vector or a *P_GAL_CIK1-CC* plasmid were released into galactose medium and incubated at 32°C. Cells were collected at the indicated time points and fixed for the examination of fluorescence signals. The relative localization of *CEN4*-GFP in cells with a normal looking metaphase spindle was determined. Of the cells with a metaphase spindle, the percentage of cells with a single GFP dot close to one spindle end was calculated and the result is shown in the left panel (n>100). The relative localization of *CEN4*-GFP signals to the spindle in some representative cells is shown in the right panel. B. *cdc13-1 MTW1-GFP TUB1-mCherry* cells with a vector or a *P_GAL_CIK1-CC* plasmid were released into galactose medium and incubated at 32°C. Cells were collected at the indicated time points. Among the cells with a metaphase spindle, the percentage of cells with two clear GFP foci was counted and the result is shown in the left panel (n>100). The spindle morphology and Mtw1-GFP distribution in some representative cells are shown in the right panel.

After the establishment of bipolar attachment, chromosomes congress to the spindle equator [37]. In budding yeast, the subsequent tension generation on chromosomes results in a transient separation of sister centromeres [38], [39]. *cdc13-1* mutant cells arrest at preanaphase at high temperatures because of the activation of the DNA damage checkpoint and bipolar attachment is believed to be established in these arrested cells [40], [41], [42]. To assay the bipolar establishment in cells lacking Cik1/Kar3, we introduced the *P_GAL_CIK1-CC* plasmid into *cdc13-1* cells with GFP-marked centromeres of chromosome IV (*CEN4*-GFP) and mCherry-labeled microtubules *(TUB1-mCherry)*. The relative localization of *CEN4*-GFP to the spindle was analyzed after G_1_ release into galactose medium at 32°C, the restrictive temperature for *cdc13-1*. Similar to *cik1Δ* mutant cells, overexpression of *CIK1-CC* also causes the formation of a dot-like spindle in some cells. Here, we only counted the cells with a metaphase spindle structure (>1.5 µM). After the establishment of bipolar attachment, we speculate that *CEN4*-GFP will either separate as two dots along the spindle or localize in the middle part of the spindle as a single dot. Nevertheless, the localization of a *CEN4*-GFP dot at the end of a spindle will suggest defective bipolar attachment. After G_1_ release for 150 min, only 12% of control cells showed a *CEN4*-GFP dot at one end of the spindle, but the percentage of cells with the GFP dot at the end of the spindle increased to 51% in those overexpressing *CIK1-CC* (Figure 3A), indicating that cells lacking Cik1/Kar3 function exhibit impaired chromosome bipolar attachment even when the spindle appears normal.

After tension establishment, kinetochores are resolved into two distinct clusters lying between the spindle poles before anaphase entry [43]. To test whether overexpression of *CIK1-CC* causes the failure of the formation of two kinetochore clusters, we introduced a *P_GAL_CIK1-CC* plasmid into *cdc13-1* strains with *TUB1-mCherry* and GFP-tagged *MTW1*, which encodes a kinetochore protein. After release from G_1_ into 32°C galactose medium for 150 min, 82% of *cdc13-1* cells with a metaphase spindle showed two clearly resolved Mtw1-GFP foci in the absence of *Cik1-CC* induction. When *CIK1-CC* was overproduced, however, only 25% of cells exhibited two clear GFP foci among the cells with a normal metaphase spindle and many cells showed scattered GFP signals along the entire spindle (Figure 3B). Together, these results strongly suggest that dysfunctional Cik1/Kar3 leads to chromosome bipolar attachment defects. Although we cannot exclude the possibility that the abnormal spindle in cells lacking Cik1/Kar3 contributes to bipolar attachment defects, our data suggest a spindle-independent role of Cik1/Kar3 in bipolar attachment.

### Cells with dysfunctional Cik1/Kar3 show syntelic attachment that allows chromosome mis-segregation in the absence of the spindle or the tension checkpoint

**Figure 4:**
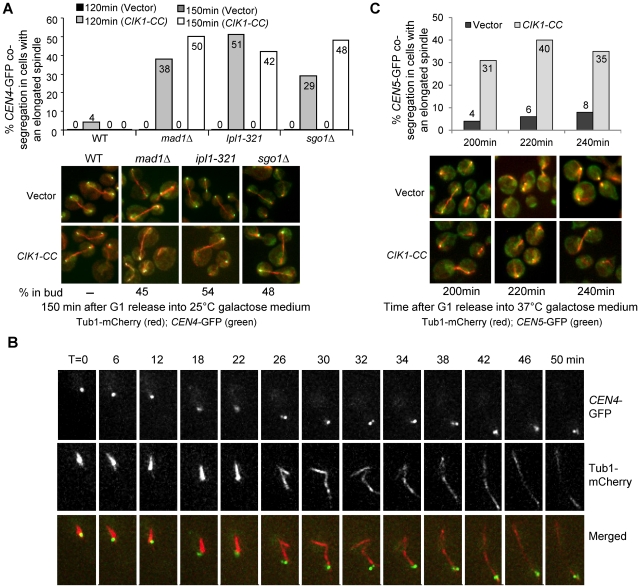
Overexpression of *CIK1-CC* results in syntelic attachment. A. Mutants in the spindle or the tension checkpoint lead to chromosome mis-segregation in cells overexpressing *CIK1-CC*. A vector or a *P_GAL_CIK1-CC* plasmid was introduced into WT, *mad1Δ*, *ipl1-321*, and *sgo1Δ* cells with *CEN4-GFP TUB1-mCherry*. The transformants were first arrested in G_1_ phase and then released into galactose medium at 25°C. Cells were collected at 120 and 150 min and fixed for the examination of fluorescence signals. The percentage of cells with mis-segregated sister *CEN4*-GFPs among those with an elongated spindle is shown in the upper panel (n>100). The localization of *CEN4*-GFP as well as spindle morphology in some representative cells is shown in the bottom panel. The numbers at the bottom of the images represent the percentage of cells that show GFP signal in the daughter cell among all of those with mis-segregated *CEN4*-GFP (n>100). B. Live-cell image of the segregation of *CEN4*-GFP in a *sgo1Δ* cell overexpressing *CIK1-CC*. *sgo1Δ CEN4-GFP TUB1-mCherry* cells with vectors or *P_GAL_CIK1-CC* plasmids were arrested in G_1_ in raffinose medium. After release into galactose medium for 2 hr, the cells were spotted onto the surface of a slide covered with agarose medium (galactose) and subjected to live-cell microscopy. At each time point, a Z-stack with 8 planes, separated by 0.5 µm, was acquired and subsequently projected. C. The segregation of *CEN5*-GFP in *mcd1-1* cohesin mutant cells after *CIK1-CC* overexpression. *mcd1-1 TUB1-mCherry CEN5-GFP* with either a vector or a *P_GAL_CIK1-CC* plasmid were arrested in G_1_ phase in raffinose medium at 25°C and then released into 37°C galactose medium. We collected cells at 200, 220, and 240 min when majority of the cells were large budded to examine the spindle morphology and the segregation of *CEN5*-GFP. The percentage of *CEN5*-GFP co-segregation in cells with an elongated spindle is shown on the top (n>100). The localization of *CEN5*-GFP as well as spindle morphology in some representative cells is shown at the bottom panel.

To further study the role of Cik1/Kar3 in chromosome bipolar attachment, we examined the chromosome segregation process in synchronized *mad1Δ* checkpoint mutant cells with *CEN4-GFP TUB1-mCherry* in the absence of Cik1/Kar3 function. After G_1_ release, *CIK1-CC* overexpression caused an obvious cell cycle delay in WT cells as indicated by the higher proportion of large budded cells, but *mad1Δ* suppressed this delay completely. Among the cells with an elongated spindle after G_1_ release for 150 min, more than 40% of *mad1Δ* cells showed *CEN4*-GFP co-segregation when Cik1-CC was overproduced, where one or two GFP dots were close to only one of the spindle poles. However, in *mad1Δ* cells with a vector control, no co-segregation of sister chromatids was observed (Figure 4A), indicating that Cik1-CC overproduction causes a kinetochore attachment defects.

The co-segregation of chromosome IV in *mad1Δ* mutant cells overexpressing *CIK1-CC* could be a consequence of syntelic attachment, monotelic attachment, or chromosome detachment. For a detached chromosome, both sister chromatids will stay in the mother cell after spindle elongation. For a chromosome with monotelic attachment, after anaphase onset the detached chromatid will stay in the mother cell but the attached one will move along with the connected spindle poles to either the mother or the daughter cell. Hence, it is impossible for both sister chromatids to move to the daughter cell together when a chromosome is either detached or with monotelic attachment. For a chromosome with syntelic attachment, however, the sister chromatids will co-segregate to either the mother or the daughter cell. Therefore, co-segregation of sister chromatids to the daughter cell will be an indication of syntelic attachment. We examined the frequency of sister-*CEN4*-GFP co-segregation into daughter cells in *mad1Δ* mutants overexpressing *CIK1-CC*. Mother cells are usually bigger in size and show a shmoo-like morphology because α-factor was used for G_1_ synchronization. Among the *mad1Δ* cells that show *CEN4*-GFP co-segregation after Cik1-CC induction, 45% of them have the GFP signal in the daughter cell (Figure 4A), indicating the presence of syntelic attachment. As this number is close to 50%, the chance of syntelic attachment to either spindle pole is similar.

If syntelic attachment in cells lacking Cik1/Kar3 function leads to a cell cycle delay, we expect that this delay depends on the tension checkpoint, because chromosomes with syntelic attachment are not under tension. Thus, we examined the chromosome segregation in *ipl1-321* and *sgo1Δ* cells at 25°C after *CIK1-CC* overexpression. Strikingly, more than 40% *ipl1-321* and *sgo1Δ* cells with an elongated spindle exhibited co-segregation of sister *CEN4*-GFPs after G_1_ release for 150 min, which is similar to *mad1Δ* checkpoint mutants. Among them, 54% of *ipl1-321* and 48% of *sgo1Δ* cells showed exclusive daughter cell localization of *CEN4*-GFP signal. In contrast, no co-segregation was observed in the mutant cells with a vector control (Figure 4A). Given the fact that the loss of function of Ipl1 or Sgo1 fails to abolish the cell cycle arrest in response to detached chromosomes [19], [20], this result further indicates that dysfunctional Cik1/Kar3 induces syntelic attachment. Since we performed the experiments at 25°C, the data demonstrate that *ipl1-321* mutant cells lose tension checkpoint function at the permissive temperature, which is in agreement with the observation that *ipl1-321* mutants exhibit reduced kinase activity at the permissive temperature [36].

We also used a live-cell imaging system to follow chromosome segregation in *sgo1Δ* mutants overexpressing *CIK1-CC* in order to clarify if some sister chromatids are connected to a single spindle pole. When incubated in galactose medium, the sister *CEN4*-GFPs migrated along with one single spindle pole and entered the daughter cell as the spindle elongated in the cell shown in Figure 4B and Video S1, indicating that both sister chromatids of chromosome IV are connected to one spindle pole. During spindle elongation, we observed separation of the two GFP dots at some time points, suggesting the absence of sister chromatid cohesion. Therefore, the sister chromatid co-segregation observed in this representative cell is likely a consequence of syntelic attachment, but not monotelic attachment. Chromosome mis-segregation was not observed in WT cells overexpressing *CIK1-CC* in this live-cell assay.

Residual cohesion may contribute to sister-chromatid co-segregation of a chromosome with monotelic attachment. To further distinguish syntelic from monotelic attachment, we examined sister-chromatid segregation in *mcd1-1* cohesin mutants while overexpressing *CIK1-CC*. When incubated at 37°C, the absence of cohesion in *mcd1-1* mutants not only abolishes the connection between sister chromatids, but also allows the spindle to elongate regardless of checkpoint activation [44]. We introduced a vector and a *P_GAL_CIK1-CC* plasmid into a *mcd1-1 CEN5-GFP TUB1-mCherry* strain. After G_1_ release into galactose medium at 37°C for 200 min, 31% of *mcd1-1* mutant cells with an elongated spindle exhibited co-segregation of sister *CEN5*-GFPs when Cik1-CC was induced. In the absence of Cik1-CC expression, however, only 4% *mcd1-1* cells with an elongated spindle showed *CEN5*-GFP co-segregation, presumably because cohesion also contributes to bipolar attachment [45] (Figure 4C). Moreover, the chance of sister-chromatid co-segregation into the mother or the daughter cell is similar. The dramatically increased frequency of sister-chromatid co-segregation in cohesin mutants after Cik1-CC overexpression further demonstrates the presence of syntelic attachment. Together, these data support the conclusion that loss of function of Cik1/Kar3 by overexpressing *CIK1-CC* causes syntelic attachment, where two sister kinetochores attach to the same spindle pole.

### A temperature sensitive mutant *kar*3-*64* shows chromosomes with syntelic attachment

**Figure 5:**
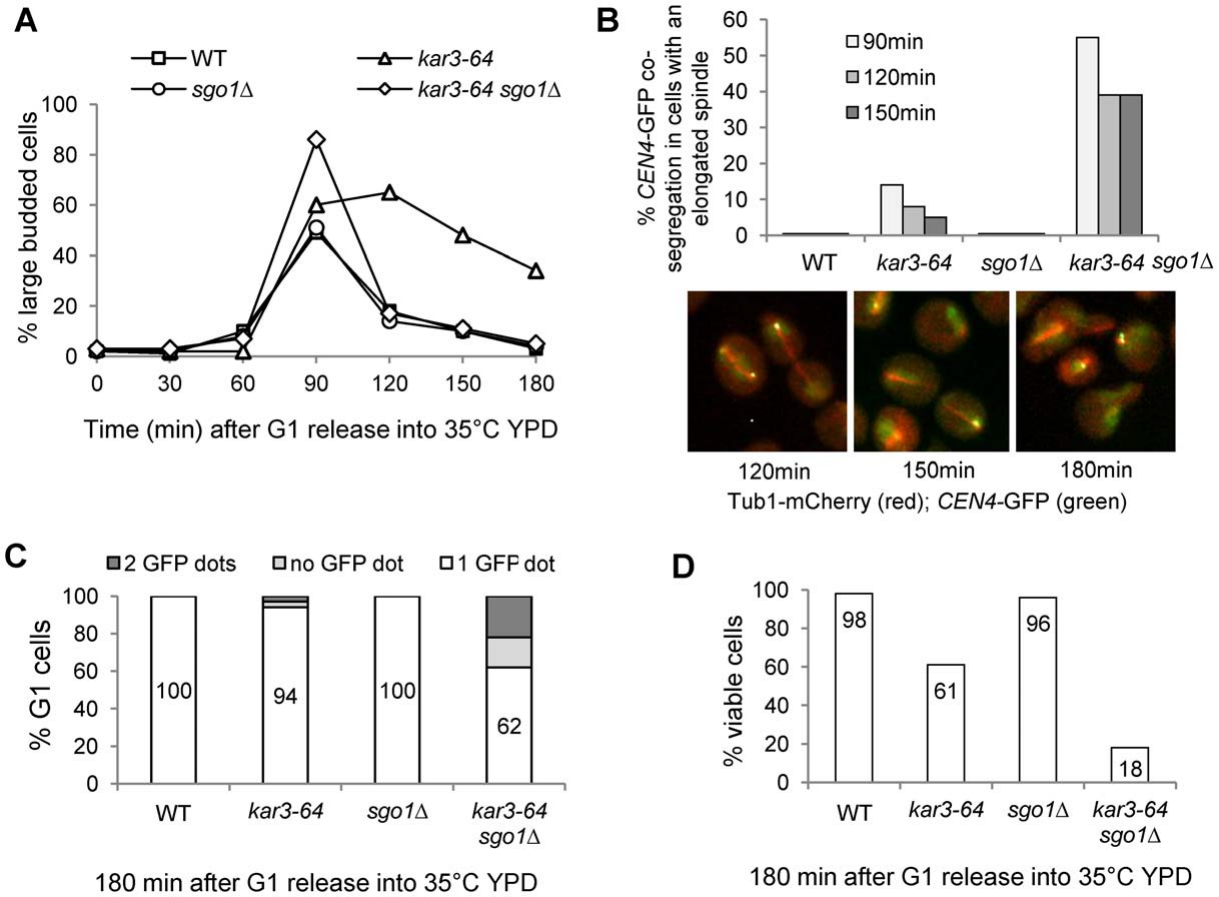
Temperature sensitive *kar3-64* mutants show syntelic attachment. G_1_-arrested WT, *kar3-64*, *sgo1Δ*, and *kar3-64 sgo1Δ* cells with *CEN4*-GFP *TUB1-mCherry* were released into YPD at 35°C. Cells were collected at the indicated time points and fixed for the examination of fluorescence signals. A. Budding index. B. The percentages of cells with mis-segregated sister *CEN4*-GFP among those with an elongated spindle (n>100) is shown in the upper panel. The *CEN4*-GFP signal and spindle morphology in some representative *kar3-64 sgo1Δ* mutant cells are shown in the bottom panel. C. Chromosome mis-segregation generates cells with zero or two *CEN4*-GFP dots after mitosis. Cells with the indicated genotypes were collected at 180 min after G_1_ release into 35°C YPD medium, and fluorescence signals were examined in cells in G_1_ phase (n>100). The percentage of cells with zero, one, or two *CEN4*-GFP dots is shown. D. *kar3-64 sgo1Δ* double mutant cells lose viability after incubation at 35°C. Cells were collected after G_1_ release for 180 min and spread onto YPD plates. After overnight incubation at 25°C, the plating efficiency was determined and the percentage of viable cells is shown (n>100).

Overexpression of the coiled-coil domain of Cik1 may disrupt the function of other proteins with a coiled-coil domain, which could also contribute to syntelic attachments. To exclude this possibility, we examined chromosome segregation in a temperature sensitive *kar3-64* mutant in the absence of Sgo1. The mutated Kar3-64 protein loses its function at high temperature because *kar3-64* is synthetically lethal with *kip3Δ* at 35°C but not at room temperature [46]. WT, *sgo1Δ*, *kar3-64*, and *kar3-64 sgo1Δ* strains with *CEN4-GFP TUB1-mCherry* were first arrested in G_1_ phase and then released into 35°C medium to inactivate Kar3. The accumulation of large budded cells in *kar3-64* mutants indicates the loss of Kar3 function (Figure 5A). Similar to the cells overexpressing *CIK1-CC*, the cell cycle delay in *kar3-64* mutant was abolished by *sgo1Δ*. We also found that about 40% of *kar3-64 sgo1Δ* double mutant cells with an elongated spindle showed *CEN4*-GFP co-segregation after release for 120 and 150 min at 35°C (Figure 5B), which is comparable to *sgo1Δ* cells overexpressing *CIK1-CC*. The majority of *kar3-64 sgo1Δ* mutant cells exited mitosis after release for 180 min. At this time point, 38% of the G_1_ cells were either absent for *CEN4*-GFP signal or showed two *CEN4*-GFP dots, suggesting the gain or loss of chromosome IV after mitosis (Figure 5C). It is also possible that some G_1_ cells have two *CEN4*-GFP dots but they are too close to be distinguished by microscopy. Consistently, only 18% of *kar3-64 sgo1Δ* mutants were viable after G_1_ release for 180 min, but 95% of WT and *sgo1Δ* cells as well as 61% of *kar3-64* cells were viable (Figure 5D). *kar3-64* cells exhibited partial viability loss presumably due to the inability to recover from mitotic arrest. These results validate the conclusion that the loss of function of Kar3 causes syntelic attachment.

The bipolar attachment defects in *kar3* mutants or in cells overexpressing *CIK1-CC* could be a result of dysfunctional Cik1/Kar3 or Vik1/Kar3, because overexpression of *CIK1-CC* also partially disrupts Vik1-Kar3 interactions (Figure S1). Moreover, previous observation that *vik1Δ* mutant is synthetically lethal with *ipl1-321* indicates a possible role of Vik1 in chromosome segregation [45]. To test whether dysfunctional Vik1 also contributes to syntelic attachment, we examined the establishment of bipolar attachment in *cdc13-1 vik1Δ* cells. Like *cdc13-1* single mutant, more than 80% of *cdc13-1 vik1Δ* cells showed either separated *CEN4*-GFP dots or one GFP dot at the center region of the spindle after G_1_ release for 90 min (Figure S2). We further examined the segregation of sister chromatids in *vik1Δ mad1Δ* double mutant cells and no mis-segregation was observed. We also crossed *vik1Δ* with *ipl1-321* and *sgo1Δ*. Surprisingly, we obtained *vik1Δ ipl1-321* and *vik1Δ sgo1Δ* double mutants and these mutants did not show co-segregation of sister chromatids (Figure S3). Therefore, *vik1Δ* mutants exhibit distinct phenotypes from *cik1Δ.* It is likely that only the Cik1/Kar3 complex is required for the establishment of chromosome bipolar attachment.

### Cik1 mediates Kar3–kinetochore interaction

**Figure 6:**
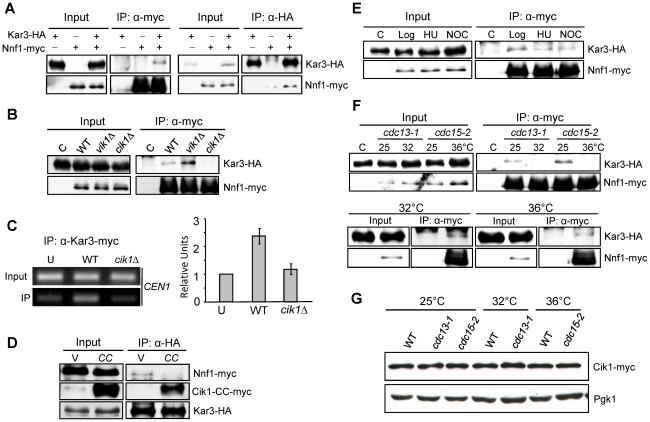
Kar3–kinetochore interaction is Cik1-dependent and cell cycle–regulated. A. Kar3 interacts with Nnf1 *in vivo*. *KAR3-3HA NNF1-13myc* cells in mid-log phase were collected to prepare cells extracts. The extracts were immunoprecipitated with either anti-HA or anti-myc antibody. The protein levels in the whole cell extracts and precipitates are shown after Western blotting with anti-HA and anti-myc antibodies. B. The interaction of Kar3 with Nnf1 depends on Cik1. WT, *cik1Δ*, and *vik1Δ* cells carrying *KAR3-3HA NNF1-13myc* were grown to mid-log phase. The extracts were immunoprecipitated with anti-myc antibody. The protein levels of Kar3-3HA and Nnf1-13myc are shown after Western blotting. C. *cik1Δ* mutant cells show deceased Kar3-centromere interaction. *KAR3-13myc* and *cik1Δ KAR3-13myc* cells in log phase were collected for ChIP assay with anti-myc antibody. The PCR products with primers specific for *CEN1* are shown in the left panel and the quantified data from three repeats are shown in the right. “U” is an untagged strain used as a control. D. Overexpression of *CIK1-CC* disrupts Kar3-Nnf1 interaction. *KAR3-3HA NNF1-13myc* cells with a vector (V) or a *P_GAL_CIK1-CC* plasmid (CC) were incubated in galactose medium for 3 hrs. The protein samples were prepared and the Kar3-Nnf1 interaction was analyzed as described in A. E. Kar3 interacts with Nnf1 in HU- and nocodazole-arrested cells. *KAR3-3HA NNF1-13myc* cells in mid-log phase were divided into three portions as untreated (Log), HU (200 mM)- and nocodazole (NOC, 20 µg/ml) - treated samples. After 3 hr treatment, the samples were analyzed for Kar3-Nnf1 interaction as described. *KAR3-3HA* cells was used as a negative control and marked as ‘C’. F. Kar3 dissociates from Nnf1 in *cdc13-1* and *cdc15-2* arrested cells. *cdc13-1* and *cdc15-2* cells with *KAR3-3HA* and *NNF1-13myc* were grown to mid-log phase at 25°C. Then temperature was shifted to 32°C (for *cdc13-1*) and 36°C (for *cdc15-2*). After 2 hr incubation, the cells were collected and the protein samples were prepared and analyzed for Kar3-Nnf1 interaction. This interaction in *cdc13-1* and *cdc15-2* mutant cells incubated at permissive and non-permissive temperatures is shown in the top panel. The Kar3-Nnf1 interaction in WT cells after incubation at 32°C and 36°C for 2 hr is shown at the bottom panel. G. The Cik1 protein levels at different cell cycle stages. WT, *cdc13-1*, and *cdc15-2* cells with *CIK1-13myc* were grown to mid-log phase at 25°C, and then shifted or 32°C (for *cdc13-1*) and 36°C (for *cdc15-2*) for 2 hrs. The cells were harvested to prepare protein samples. Cik1 protein levels are shown after Western blotting. Pgk1 proteins level was used as a loading control.

Cik1 and Vik1 are the two Kar3 partners in budding yeast and Cik1/Kar3 localizes along the length of the spindle, and probably at interpolar microtubule plus ends [25], [33]. Kar3 was also found to bind to the kinetochore to promote its transport along astral microtubules towards spindle poles [26]. The similar mitotic defects in *cik1* and *kar3* mutants suggest that Cik1 likely mediates the association of Kar3 with kinetochores. Based on the genome-wide yeast two-hybrid assay, Kar3 was shown to interact with Nnf1, a kinetochore protein in the MIND complex, [7], [47], [48]. Using a co-immunoprecipitation (co-IP) approach we found that Nnf1-myc was able to pull down Kar3-HA, and vice versa, confirming the Kar3-kinetochore interaction *in vivo*, although it remains inconclusive whether this Kar3-Nnf1 interaction is direct (Figure 6A). Then, we examined Kar3-Nnf1 interaction in the absence of either Cik1 or Vik1. As shown in Figure 6B, deletion of *CIK1* but not *VIK1* abolished this interaction completely, suggesting that the Kar3-kinetochore interaction is dependent on Cik1. Interestingly, this interaction was obviously increased in *vik1Δ* mutant cells, presumably because more Kar3 protein is available for the binding to kinetochores. Consistently, chromatin immunoprecipitation (ChIP) data shows diminished Kar3-centromere association in *cik1Δ* cells (Figure 6C). If Cik1 mediates Kar3-Nnf1 interaction, the overexpression of the coiled-coil domain of Cik1 should disrupt this interaction, because *CIK1-CC* overexpression disrupts Cik1-Kar3 interaction (Figure 1A). Indeed, Kar3-HA failed to pull down Nnf1-myc in cells overexpressing *CIK1-CC* (Figure 6D).

Data from the Sorger lab suggest that Kar3 may associate with detached kinetochores [43]. Moreover, Kar3 is essential for the lateral sliding of chromosomes towards spindle poles during S-phase [27]. Therefore, the association of Kar3 with kinetochores might be cell cycle regulated. To test this possibility, we compared Kar3-Nnf1 interaction in different cell cycle stages. Kar3 interacted with Nnf1 in both HU- and nocodazole-arrested cells (Figure 6E), when bipolar attachment has not established yet. *cdc13-1* mutant cells arrest at preanaphase with established bipolar attachment [40]. Interestingly, the Kar3-Nnf1 interaction was not detected in *cdc13-1* mutant cells after 2 hr incubation at 32°C (Figure 6F). Similarly, Kar3 did not associate with Nnf1 in *cdc15-2*-arrested telophase cells. The decreased Kar3-Nnf1 interaction in *cdc13-1* or *cdc15-2* mutant cells could be due to the degradation of Cik1 [49], thus the Cik1 protein levels were examined in WT, *cdc13-1*, and *cdc15-2* mutant cells incubated at permissive or no-permissive temperatures. It is clear that the mutant cells exhibit Cik1 protein levels comparable to WT cells when incubated at high temperatures (Figure 6G). The results suggest that the Cik1/Kar3 complex associates with kinetochores before the establishment of bipolar attachment. This association might be essential for chromosome transport as well as the achievement of bipolar attachment, but lack of this mechanism will contribute to syntelic attachment.

### The Cik1/Kar3 complex is required for efficient DASH–centromere binding

**Figure 7:**
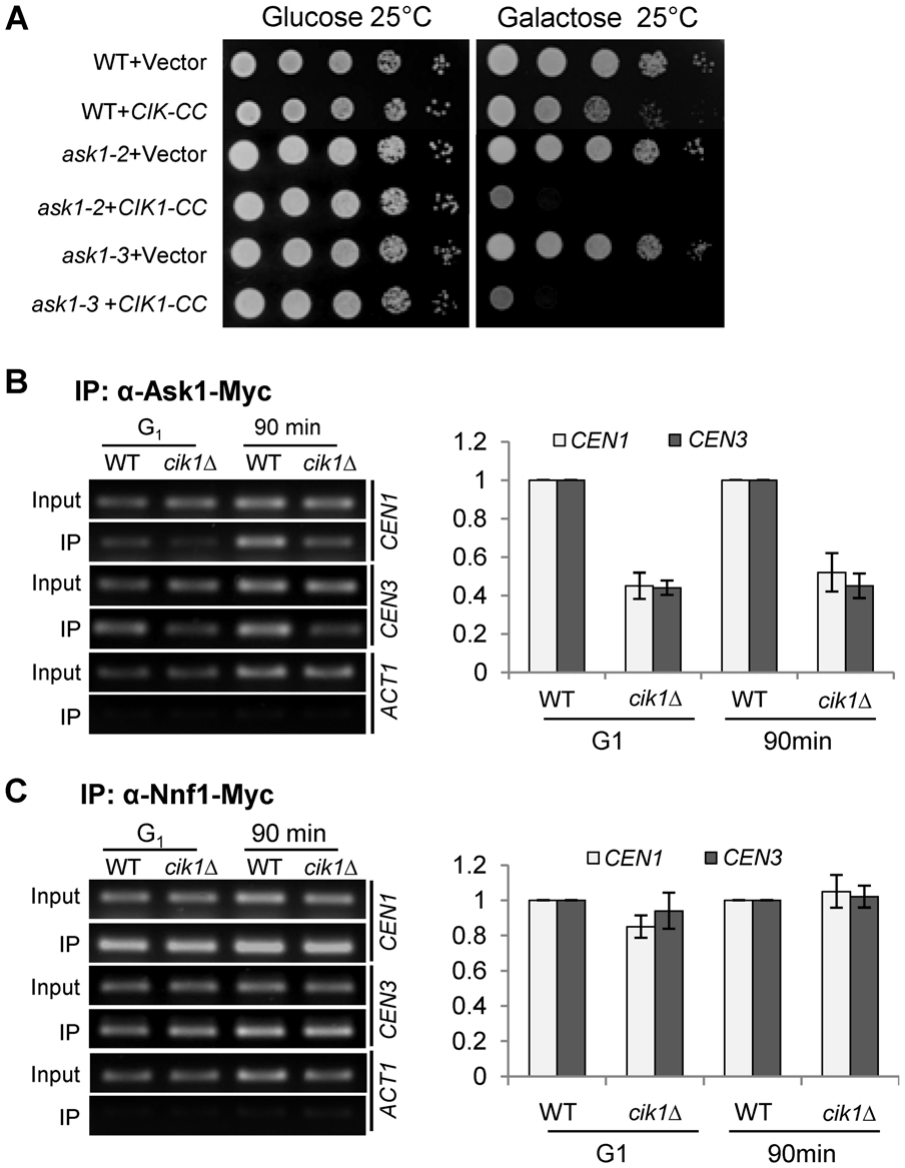
Dysfunctional Cik1/Kar3 leads to decreased DASH–kinetochore interaction. A. Overexpression of *CIK1-CC* is lethal to *ask1* mutants. Saturated cell cultures with indicated genotypes were 10-fold diluted and then spotted onto dropout plates containing glucose or galactose. B. *cik1Δ* mutant cells show decreased centromere association of Ask1. G_1_-arrested *cdc13-1 ASK1-9myc* and *cdc13-1 cik1Δ ASK1-9myc* were released to YPD medium at 34°C. The cells were collected at time 0 and 90 min for ChIP assay with anti-myc antibody. The PCR products with primers specific for *CEN1*, *CEN3*, and *ACT1* are shown in the left panel and the quantified data are shown in the right. The ratio of the PCR yield (IP/input) in WT cells was standardized as one. The experiment was repeated three times. C. *cik1Δ* mutant cells exhibit normal association of Nnf1 with centromeric DNA. *cdc13-1 NNF1-13myc* and *cdc13-1 cik1Δ NNF1-13myc* cells were treated as in B. The PCR products with primers specific for *CEN1*, *CEN3* and *ACT1* are shown in the left panel and the quantified data are shown in the right. The experiment was repeated for three times.

The DASH kinetochore complex contains 10 protein subunits including Dam1 and Ask1. Unlike other kinetochore proteins, the association of the DASH complex with kinetochores occurs only after kinetochore-microtubule interaction [2]. Interestingly, *kar3Δ* has been shown to be synthetically lethal with *dam1-1* mutant [50]. We found that overexpression of *CIK1-CC* caused lethality to *ask1-2* and *ask1-3* mutants (Figure 7A). One possibility is that the Cik1/Kar3 complex promotes bipolar attachment by inducing the DASH-kinetochore interaction. Therefore, we performed ChIP assays to examine DASH-centromere interaction in synchronous cell cultures. *cdc13-1* mutants were used to arrest cells at preanaphase, when DASH complexes have already been loaded onto centromeres [2]. Interestingly, *cdc13-1 cik1Δ* cells exhibited reduced Ask1-centromere interaction in G_1_ phase as well as after G_1_ release for 90 min (Figure 7B). In order to determine whether the Ask1 binding defect is a result of impaired kinetochore integrity, we also compared the centromere binding of another kinetochore protein Nnf1. In contrast to Ask1, the association of Nnf1 with centromeric DNA was similar in synchronous *cdc13-1* and *cdc13-1 cik1Δ* cells either before or after G_1_ release (Figure 7C), indicating that the core kinetochore structure is intact. These results suggest that the Cik1/Kar3 complex facilitates the association of the DASH complex with the core kinetochore proteins. It is our future interest to determine if decreased DASH-kinetochore interaction is the cause or a consequence of syntelic attachments.

## Discussion

In budding yeast, Kar3 is the only kinesin with minus-end-directed motor activity and the interaction with its partners, Cik1 and Vik1, is essential for its motor activity. Our data clearly demonstrate the presence of syntelic attachments in cells lacking Cik1/Kar3 function based on the following observations: first, in the absence of the spindle checkpoint, the loss of function of Cik1/Kar3 induced significant sister-chromatid co-segregation into the daughter cell. Moreover, a dysfunctional tension checkpoint is sufficient to enable anaphase entry as well as co-segregation of sister chromatids in cells lacking Cik1/Kar3 function. Because it is well established that cells with tensionless chromosomes require the tension checkpoint for cell cycle arrest, this result indicates the presence of chromosomes with syntelic attachment in cells lacking functional Cik1/Kar3. Furthermore, our live-cell imaging data show the migration of both sister chromatids along with one spindle pole during anaphase in *sgo1Δ* cells overexpressing *CIK1-CC*. As recent evidence indicates that residual sister chromatid cohesion remains during early anaphase [51], one can argue that the sister chromatid co-segregation is a consequence of monotelic attachment, where only one of the sister kinetochores connects to the spindle pole but the other co-migrates with the sister because of the residual cohesion. Our data suggest that this scenario is unlikely, because we detected separated *CEN4*-GFP dots during anaphase in a *sgo1Δ* cell overexpressing *CIK1-CC*, indicating the absence of cohesion. In addition, we found that dysfunctional Cik1/Kar3 also induces sister chromatid co-segregation in cohesin mutant cells. Therefore, we conclude that inactivation of the Cik1/Kar3 complex induces syntelic attachment.

In cells overexpressing the coiled-coil domain of *CIK1*, we observed delayed Pds1 degradation, indicating the activation of the spindle checkpoint. However, either *ipl1-321* or *sgo1Δ* abolished this delay completely. In addition, both *ipl1-321* and *sgo1Δ* mutants show normal timing of spindle elongation when *CIK1-CC* is overexpressed, resulting in a high frequency of sister-chromatid co-segregation that is comparable to *mad1Δ* mutants. Previous data indicate that *ipl1* mutants, but not *sgo1*, generate detached chromosomes when these chromosomes are not under tension [22], thus it is speculated that Ipl1 activates the spindle checkpoint by generating detached chromosomes when tension is absent. However, the complete loss of checkpoint function in *sgo1* mutant cells overexpressing *CIK1-CC* suggests that the generation of detached chromosomes is not necessary to activate the spindle checkpoint in the presence of syntelic attachments. Interestingly, we found that *ipl1-321* mutants show checkpoint defect even when grown at 25°C. Therefore, the decreased kinase activity in *ipl1-321* mutants at the permissive temperature may be unable to execute the checkpoint function, but is sufficient to support normal chromosome segregation.

Although our data indicate that cells lacking functional Cik1/Kar3 show syntelic attachments, the molecular function of this motor complex in chromosome bipolar attachment remains elusive. One possible explanation is that the Kar3-dependent poleward chromosome movement facilitates chromosome bipolar attachment. After the initial chromosome capture, Cik1/Kar3-mediated sliding of this chromosome along spindle microtubules will orient sister kinetochores so that the detached kinetochore will face the opposite spindle pole, thereby facilitating bipolar attachment. In agreement with this possibility, we found that Cik1 mediates the association of Kar3 with kinetochores and this association only occurs before chromosome bipolar attachment. In mammals and flies, kinetochore dynein mediates the poleward chromosome movement, which may also facilitate the correct orientation of sister kinetochores through a similar mechanism [52], [53]. Another possibility is that the abnormal spindle structure in *cik1* and *kar3* mutant cells may contribute to the high frequency of syntelic attachments. At 37°C, *cik1Δ* and *kar3Δ* mutants arrest with a dot-like spindle structure and many mutant cells show spindle defects even when incubated at room temperature [23], [29], [33]. We examined chromosome bipolar attachment in cells arrested at preanaphase and found that the disruption of Cik1/Kar3 function by overexpressing *CIK1-CC* increases the chance of co-localization of *CEN4*-GFP with one spindle pole in cells with a metaphase spindle that appears normal. This result suggests that the bipolar attachment defect in cells lacking Cik1/Kar3 function could be independent of the spindle defect. To separate the spindle and kinetochore functions of Cik1/Kar3, we need to identify the kinetochore protein that directly binds to the Cik1/Kar3 complex and define the domain responsible for this interaction. Mutation of this domain may selectively disrupt the kinetochore function of Cik1/Kar3 but maintain its spindle function.

In mammalian cells, syntelic attachment can be induced by treatment with a small molecule monastrol, which inhibits the activity of a kinesin Eg5 [54], [55]. Moreover, the treatment of mammalian cells with a low-dose of taxol will prevent tension generation on chromosomes [56]. In budding yeast, two methods have been widely used to introduce a tension defects for the study of the tension sensing mechanism. One is to completely block DNA replication where the absence of sister chromatids prevents tension generation, while another method is to abolish sister chromatid cohesion by using temperature sensitive *mcd1/scc1* mutants or by expressing *MCD1* from a galactose inducible promoter [19], [20]. The disadvantage of these methods is that Pds1 protein level is the only marker to monitor the checkpoint activity. Because the lack of sister-chromatid cohesion allows the spindle to elongate regardless of checkpoint activation, we cannot use spindle elongation to monitor anaphase entry. Moreover it is difficult to analyze the role of the tension checkpoint in faithful chromosome segregation. Another disadvantage is that these methods will kill the cells after the induction of tension defects, thus it is impossible to perform a genetic screen for additional genes that are required for survival in the presence of tension defects. Here we report a new approach to induce syntelic attachments by inactivating the Cik1/Kar3 motor complex, which prevents tension generation on chromosomes but maintains intact kinetochore attachment. We have demonstrated that overexpression of the coiled-coil domain of Cik1 from a GAL promoter disrupts Cik1-Kar3 interaction, which allows us to conditionally induce syntelic attachment by growing cells with *P_GAL_CIK1-CC* plasmid in galactose medium. This approach will be a critical tool to study the response to tension defects in budding yeast.

## Materials and Methods

### Strains, plasmids, and growth conditions

The strains used in this study are derivatives of W303 and listed in Table S1. Gene deletions and epitope tagging were performed by using a PCR-based protocol [57]. The *P_GAL_CIK1-CC* plasmid was constructed by inserting the *CIK1* coiled-coil fragment into a *CEN-TRP-GAL-myc* vector.

To arrest yeast cells in G_1_ phase, 5 µg/ml α-factor was added into mid-log phase cells grown in YPD or in TRP dropout medium containing 2% raffinose at 25°C for 2.5 hr. G_1_-arrested cells were centrifuged and washed twice with water to release into YPD at 32°C for *cdc13-1* arrest or TRP dropout medium containing 2% galactose at 25°C for *CIK1-CC* overexpression. To block the next cell cycle, 15 µg/ml α-factor was added when majority of the cells were budded. Hydroxyurea was purchased from ACROS Organics and the final concentration was 100 mM for HU plates.

### Protein techniques

The yeast protein samples were separated and detected as described previously [58]. Protein samples were prepared using an alkaline method and were resolved by 10% SDS- PAGE. Primary antibodies (anti-myc and anti-HA) were purchased from Covance (Madison, WI), and anti-Pgk1 antibody was from Molecular Probes (Eugene, OR). The HRP-conjugated secondary antibody was purchased from Jackson ImmunoResearch (West Grove, PA).

### Fluorescence microcopy

Cells were collected and fixed with 3.7% formaldehyde for 15 min at room temperature. The cells were washed once with 1×PBS (pH7.2) and then resuspended in 1×PBS buffer to examine fluorescence signals with a microscope (Zeiss Axioplan 2).

### Co-immunoprecipitation (co-IP) and chromatin immunoprecipitation (ChIP) assay

Cell cultures were collected and washed once with water. After being resuspended in RIPA buffer (25 mM Tris PH7.5, 10 mM EDTA, 150 mM NaCl and 0.05% Tween-20) supplied with protease inhibitors, cells were homogenized with a bead-beater. The resulting cell extracts were incubated with primary antibody overnight at 4°C. The cell extracts were then incubated with protein-A conjugated agarose beads (Santa Cruz Biotechnology), which was pre-incubated with BSA at 4°C. After incubation for 1 hr, the beads were collected by centrifugation and washed with RIPA buffer for three times. Equal volume RIPA and protein loading buffer were added and the protein samples were boiled for 5 min for Western blot analysis. The ChIP assay was performed as described previously [59].

### Live-cell fluorescence microscopy

For live-cell microscopy, we used a concave glass slide as a culture chamber, which was filled with 2% agarose dissolved in galactose medium. The agarose pad was solidified for 5 min at room temperature before use. Cells were first arrested in G_1_ phase in raffinose medium. After release into galactose medium for 2 hr, 1.5 µl concentrated cells were laid on the top of the agarose pad, which was then sealed with a piece of cover glass. Live-cell microscopy was carried out on a DeltaVision imaging system equipped with an environmental chamber (Applied Precision, Inc.). All live-cell images were acquired at 25°C with a 100× (NA = 1.41) objective lens on an Olympus ix71 microscope. A total of 8 z-stacks were collected at each time point and each optical section was 0.5 µm thick. Exposure time for each optical section was set between 60 and 100 ms and the time-lapse interval was set at 2 min. Projected images were used for display.

## Supporting Information

Figure S1 Overexpression of *CIK1-CC* decreases Kar3-Vik1 interaction. *KAR3-3HA VIK1-13myc* cells with a vector or a *P_GAL_CIK1-CC* plasmid were grown to mid-log phase in raffinose medium at 30°C. After 2% galactose was added to the cell cultures to for 3 hr, the cells were collected for immunoprecipitation assay with anti-HA antibody and the precipitates were subjected to Western blotting following SDS-PAGE.
(TIF)

Figure S2 The chromosome bipolar attachment is normal in *vik1Δ* mutant cells. G_1_-arrested *cdc13-1 CEN4-GFP TUB1-mCherry* and *vik1Δ cdc13-1 CEN4-GFP TUB1-mCherry* cells were released into YPD medium at 32°C. Cells were collected at the indicated time points and fixed for the examination of fluorescence signals. The relative localization of *CEN4*-GFP to the metaphase spindle was determined. The percentage of cells with separated *CEN4*-GFP dots or with a *CEN4*-GFP dot localized at the middle part of the spindle is shown in A. The spindle morphology and *CEN4*-GFP distribution in some representative cells are shown in B.
(TIF)

Figure S3 *vik1Δ* cells exhibit normal sister chromatid segregation in the absence of the spindle or the tension checkpoint. *vik1Δ* single and *vik1Δ mad1Δ*, *vik1Δ sgo1Δ*, *vik1Δ ipl1-321* double mutants with *TUB1-mCherry CEN4-GFP* were arrested in G_1_ phase and then released into YPD medium at 25°C. Cells were collected for the budding index and the examination of *CEN4-GFP* segregation. The budding index is shown in the top panel; the localization of *CEN4*-GFP and spindle morphology are shown in the bottom panel.
(TIF)

Table S1 Strains used in this study.
(DOCX)

Video S1 The co-segregation of sister *CEN4*-GFP in a *sgo1Δ* cell overexpressing *CIK1-CC*. *sgo1Δ CEN4-GFP TUB1-mCherry* cells with *P_GAL_CIK1-CC* plasmids were arrested in G_1_ phase in raffinose medium. After release into galactose medium for 2 hr, the cells were laid onto the surface of an agarose pad (galactose medium) and subjected to live-cell microscopy. Every 2 min, a Z-stack with 8 planes, separated by 0.5 µm, was acquired and subsequently projected.
(AVI)
