# Supplementary figures and images for: Loss of Function of the Cik1/Kar3 Motor Complex Results in Chromosomes with Syntelic Attachment That Are Sensed by the Tension Checkpoint
Source: PLoS Genet. 2012 Feb 2;8(2):e1002492. doi: 10.1371/journal.pgen.1002492 (PMC3271067; doi:10.1371/journal.pgen.1002492)

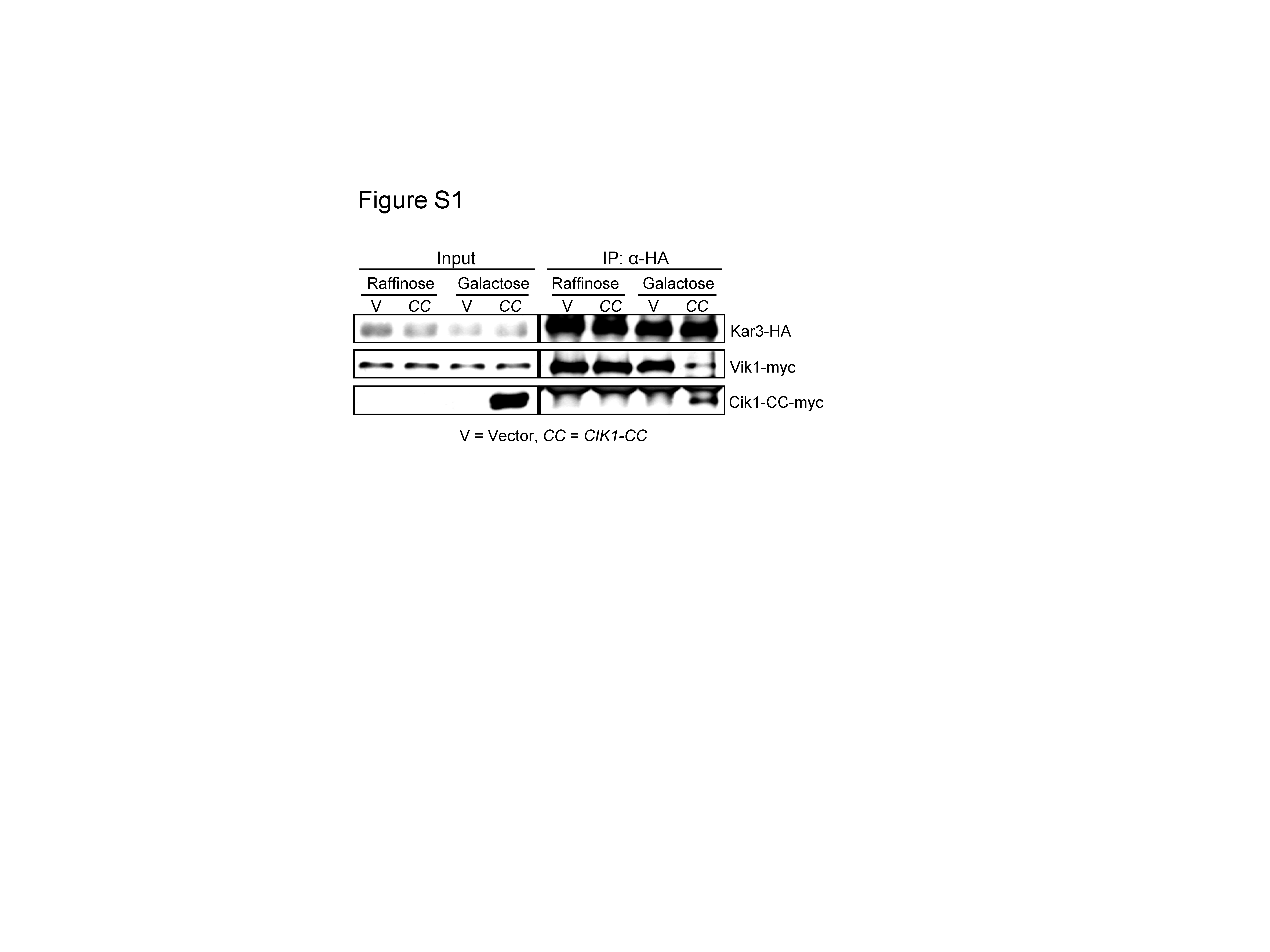

Supplement: Figure S1 — Overexpression of CIK1-CC decreases Kar3-Vik1 interaction. KAR3-3HA VIK1-13myc cells with a vector or a PGALCIK1-CC plasmid were grown to mid-log phase in raffinose medium at 30°C. After 2% galactose was added to the cell cultures to for 3 hr, the cells were collected for immunoprecipitation assay with anti-HA antibody and the precipitates were subjected to Western blotting following SDS-PAGE. (TIF) [file pgen.1002492.s001.tif]

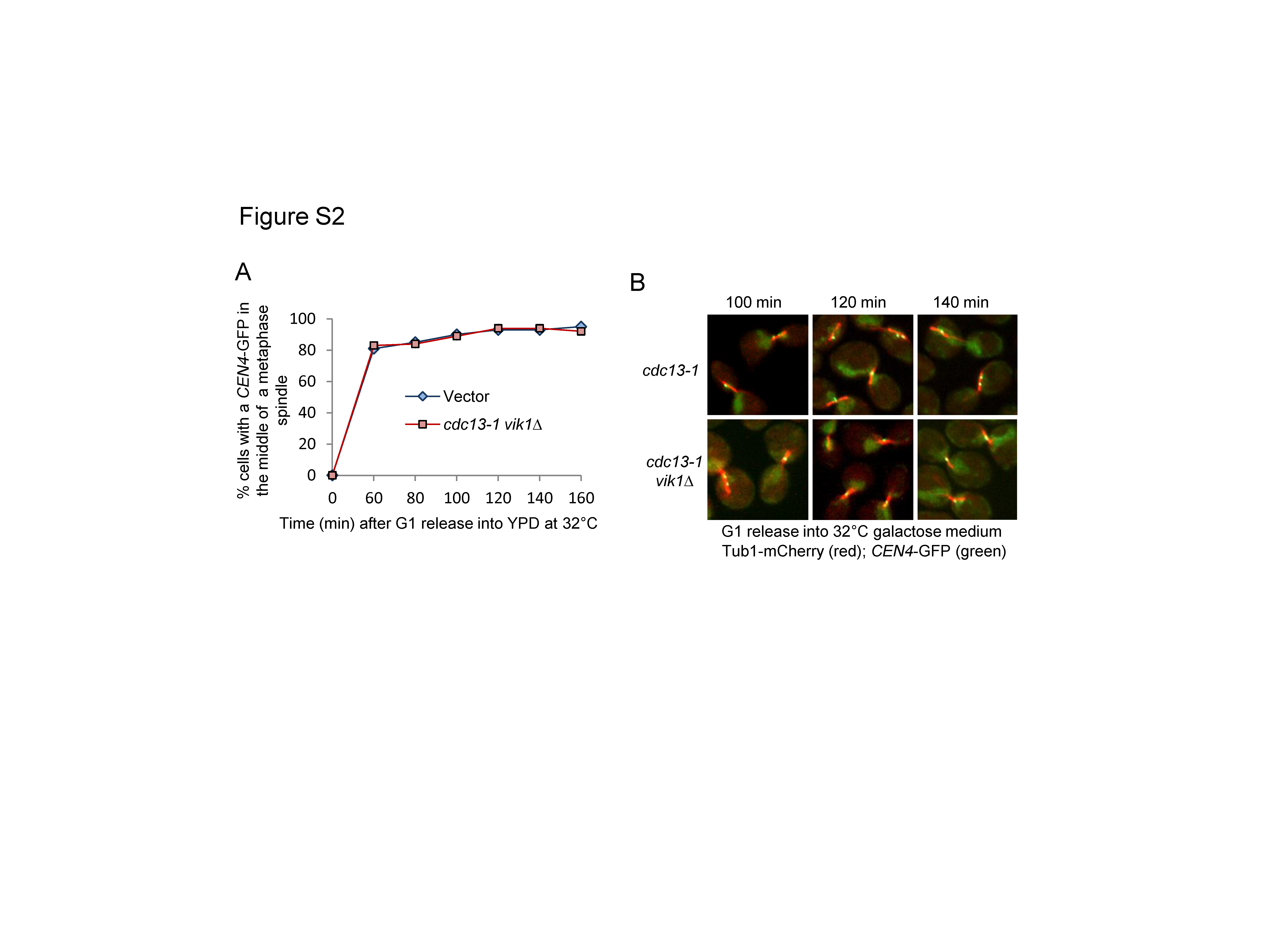

Supplement: Figure S2 — The chromosome bipolar attachment is normal in vik1Δ mutant cells. G1-arrested cdc13-1 CEN4-GFP TUB1-mCherry and vik1Δ cdc13-1 CEN4-GFP TUB1-mCherry cells were released into YPD medium at 32°C. Cells were collected at the indicated time points and fixed for the examination of fluorescence signals. The relative localization of CEN4-GFP to the metaphase spindle was determined. The percentage of cells with separated CEN4-GFP dots or with a CEN4-GFP dot localized at the middle part of the spindle is shown in A. The spindle morphology and CEN4-GFP distribution in some representative cells are shown in B. (TIF) [file pgen.1002492.s002.tif]

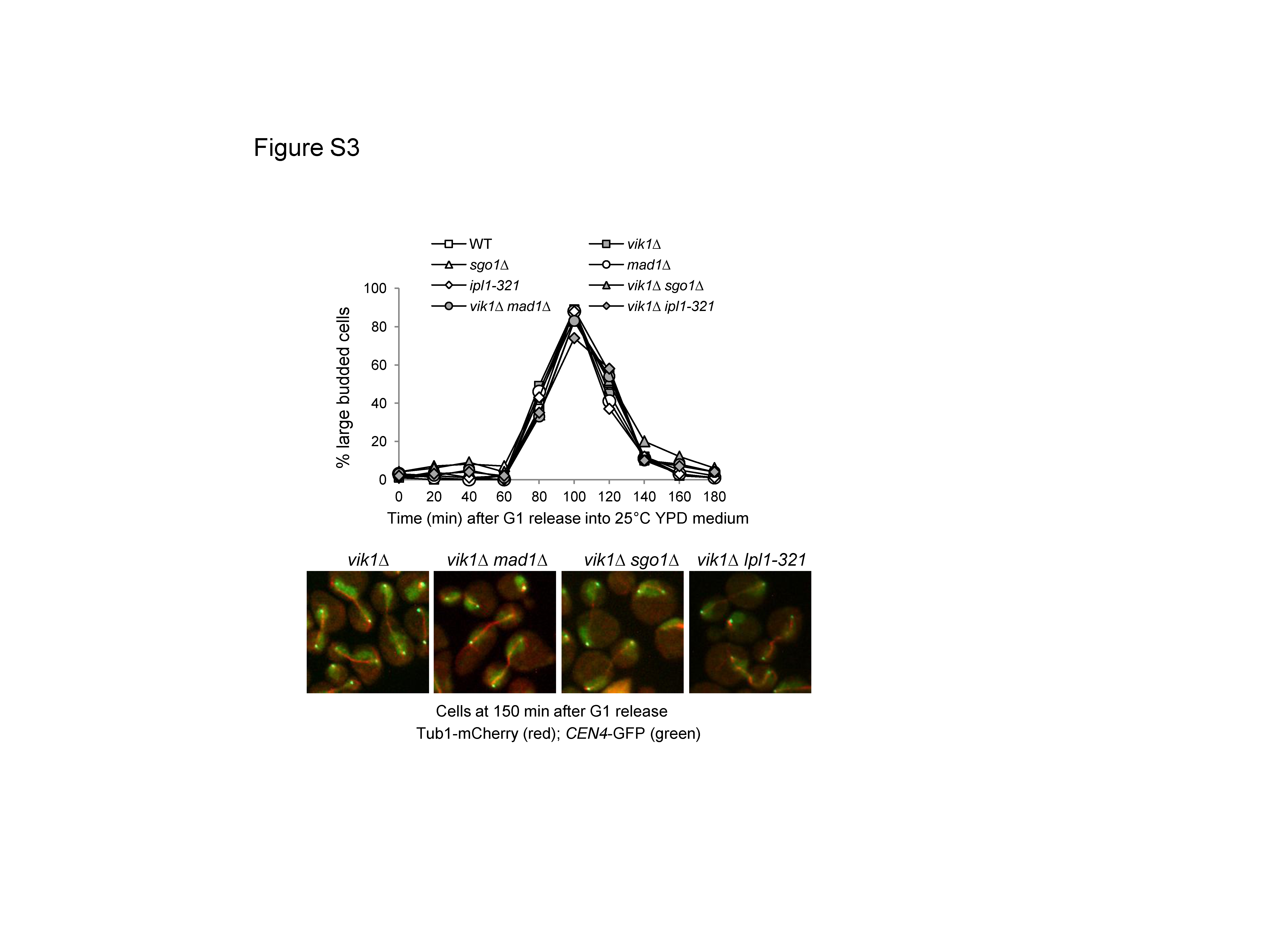

Supplement: Figure S3 — vik1Δ cells exhibit normal sister chromatid segregation in the absence of the spindle or the tension checkpoint. vik1Δ single and vik1Δ mad1Δ, vik1Δ sgo1Δ, vik1Δ ipl1-321 double mutants with TUB1-mCherry CEN4-GFP were arrested in G1 phase and then released into YPD medium at 25°C. Cells were collected for the budding index and the examination of CEN4-GFP segregation. The budding index is shown in the top panel; the localization of CEN4-GFP and spindle morphology are shown in the bottom panel. (TIF) [file pgen.1002492.s003.tif]
