# Supplementary material for: Loss of Function of the Cik1/Kar3 Motor Complex Results in Chromosomes with Syntelic Attachment That Are Sensed by the Tension Checkpoint
Source: PLoS Genet. 2012 Feb 2;8(2):e1002492. doi: 10.1371/journal.pgen.1002492 (PMC3271067; doi:10.1371/journal.pgen.1002492)
Supplement: Table S1 — Strains used in this study. (DOCX) [file pgen.1002492.s004.docx]

**Table S1.** **Strains used in this study**

| **Strains** | **Relevant Genotypes** | **Reference** |
| --- | --- | --- |
| Y300 | *MAT***a** *ura3-1 his3-11,15 leu2-3,112 trp1-1 ade2-1 can1-100* | Lab stock |
| 948-3-1 | *MAT***a** *CIK1-13myc::Sphis5^+^ KAR3-3HA::Sphis5^+^* | This study |
| 797-19-4 | *MAT***a** *ipl1-321* | Biggins lab |
| YYW187 | *MAT***a** *mad1Δ::HIS3* | This study |
| YYW102 | *MAT***a** *sgo1Δ::KanMX* | This study |
| 300-1-1 | *MAT***a**  *PDS1-18myc::LEU2* | This study |
| 911-5-1 | *MAT***a** *ipl1-321 PDS1-18myc::LEU2* | This study |
| 771-4-1 | *MAT***a** *mad1Δ::HIS3 PDS1-18myc::LEU2* | This study |
| 2024-9-3 | *MAT***a** *sgo1Δ::KanMX PDS1-18myc::LEU2* | This study |
| YYW141 | *MAT***a** *promURA3::tetR::GFP-LEU2 CENIV::tetOX448::URA3 TUB1-mCherry::URA3* | This study |
| 1091-5-3 | *MAT***a** *cdc13-1 promURA3::tetR::GFP-LEU2 CENIV::tetOX448::URA3 TUB1-mCherry::URA3* | This study |
| 1092-9-2 | *MAT***a** *cdc13-1 MTW1-3GFP::HIS3 TUB1-mCherry::URA3* | This study |
| 2371-1-3 | *MAT***a** *ipl1-321 promURA3::tetR::GFP::LEU2 CENIV::tetOX448::URA3 TUB1-mCherry::URA3* | This study |
| 2378-2-1 | *MAT***a** *sgo1Δ::KanMX promURA3::tetR::GFP::LEU2 CENIV::tetOX448::URA3 TUB1-mCherry::URA3* | This study |
| 2370-1-4 | *MAT***a** *mad1Δ::HIS3 promURA3::tetR::GFP::LEU2 CENIV::tetOX448::URA3 TUB1-mCherry::URA3* | This study |
| 2624-7-1 | *MAT***a** *vik1Δ:: Sphis5^+^ promURA3::tetR::GFP::LEU2 CENIV::tetOX448::URA3 TUB1-mCherry::URA3* | This study |
| 2624-1-1 | *MAT***a** *vik1Δ::HIS3 sgo1Δ::KanMX promURA3::tetR::GFP::LEU2 CENIV::tetOX448::URA3 TUB1-mCherry::URA3* | This study |
| 2630-6-2 | *MAT***a** *vik1Δ::Sphis5^+^ mad1Δ::HIS3 promURA3::tetR::GFP::LEU2 CENIV::tetOX448::URA3 TUB1-mCherry::URA3* | This study |
| 2631-5-2 | *MAT***a** *vik1Δ::Sphis5^+^ ipl1-321 promURA3::tetR::GFP::LEU2 CENIV::tetOX448::URA3 TUB1-mCherry::URA3* | This study |
| 2667-1-4 | *MAT***a** *mcd1-1 trp1:: Sphis5^+^* tetR-GFP::LEU2 tetO-CEN5::HIS3*TUB1-mCherry::URA3* | This study |
| 2508-3-1 | *MAT***a** *promURA3::tetR::GFP-LEU2 CENIV::tetOX448-URA3 TUB1-mCherry::URA3 (CEN-HIS3-kar3-64)* | This study |
| 2508-6-3 | *MAT***a** *kar3Δ::TRP1 promURA3::tetR::GFP::LEU2 CENIV::tetOX448::URA3 TUB1-mCherry::URA3 (CEN-HIS3-kar3-64)* | This study |
| 2507-8-2 | *MAT***a** *sgo1Δ::KanMX promURA3::tetR::GFP::LEU2 CENIV::tetOX448::URA3 TUB1-mCherry::URA3 (CEN-HIS3-kar3-64)* | This study |
| 2507-1-1 | *MAT***a** *sgo1Δ::KanMX kar3Δ::TRP1 promURA3::tetR::GFP::LEU2 CENIV::tetOX448::URA3 TUB1-mCherry::URA3 (CEN-HIS3-KAR3-64)* | This study |
| 756-9-2 | *MAT***a** *NNF1-13myc::KanMX* | This study |
| YYW143 | *MATa KAR3-3HA::Sphis5^+^* | This study |
| 766-4-2 | *MATa KAR3-3HA::Sphis5^+^ NNF1-13myc::KanMX* | This study |
| 780-5-1 | *MATa cik1Δ::KanMX NNF1-13myc::KanMX* | This study |
| YYW159 | *MATa CIK1-13myc::Sphis5^+^* | This study |
| 2625-3-2 | *MATa cdc13-1 CIK1-13myc::Sphis5^+^* | This study |
| 2626-1-1 | *MATa cdc15-2 CIK1-13myc::Sphis5^+^* | This study |
| 2632-5-4 | *MATa cdc13-1 NNF1-13myc::KanMX* | This study |
| 2632-4-3 | *MATa cdc13-1 cik1Δ::KanMX NNF1-13myc::KanMX* | This study |
| 1030-1-1 | *MATa cik1Δ::KanMX KAR3-3HA::Sphis5^+^ NNF1-13myc::KanMX* | This study |
| 1030-4-3 | *MATa vik1Δ::KanMX KAR3-3HA::Sphis5^+^ NNF1-13myc::KanMX* | This study |
| 1051-9-1 | *MATa cdc13-1 KAR3-3HA::Sphis5^+^ NNF1-13myc::KanMX* | This study |
| 1052-1-1 | *MATa cdc15-2 KAR3-3HA::Sphis5^+^ NNF1-13myc::KanMX* | This study |
| 854-2-3 | *MATa ask1-2* | Elledge lab |
| Y1103 | *MATa ask1-3* | Elledge lab |
| 867-23-1 | *MATa cdc13-1 ASK1-9myc::HIS3* | This study |
| 867-24-1 | *MATa cdc13-1 cik1Δ::KanMX ASK1-9myc::HIS3* | This study |
